# Open pilot study of a guided digital self-help intervention targeting sleep and the biological clock in university students using a pre-test post-test design

**DOI:** 10.1038/s41598-025-04891-8

**Published:** 2025-07-01

**Authors:** Laura M. Pape, Niki Antypa, Philip Spinhoven, Annemieke van Straten, Sascha Y. Struijs, Caring Universities Consortium, Sascha Struijs, Nadia Garnefski, Vivian Kraaij, Elske Salemink, Petra Hurks, Marilisa Boffo, Danielle Remmerswaal, Reinout Wiers, Claudia van der Heijde, Lisa Klinkenberg, Monique de Bruijn-Smolders, Jessica Nooij

**Affiliations:** 1 Department of Clinical Psychology, Leiden University, Leiden, The Netherlands; 2 Department of Clinical, Neuro- and Developmental Psychology, VU University Amsterdam, Amsterdam, The Netherlands; 3 Amsterdam Public Health Research Institute, Vrije Universiteit Amsterdam, Amsterdam, The Netherlands; 4 Vrije Universiteit Amsterdam, Amsterdam, The Netherlands; 5 Leiden University, Leiden, The Netherlands; 6 Utrecht University, Utrecht, The Netherlands; 7 Maastricht University, Maastricht, The Netherlands; 8 Erasmus University Rotterdam, Rotterdam, The Netherlands; 9 University of Amsterdam, Amsterdam, The Netherlands; 10 InHolland University of Applied Sciences, Hoofddorp, The Netherlands; 11 Rotterdam University of Applied Sciences, Rotterdam, The Netherlands; 12 Avans University of Applied Sciences, Breda, The Netherlands

**Keywords:** E-health, Intervention, Sleep, Biological clock, CBT-I, University students, Psychology, Disease prevention

## Abstract

Insomnia is common among university students and is associated with mental health problems. Students often have irregular day/night rhythms, which contribute to circadian rhythm disruptions. This open pilot study investigated the feasibility, acceptability, and preliminary effectiveness of a 5-week digital guided self-help intervention in university students with self-reported insomnia. The intervention is based on cognitive behavioural therapy for insomnia with specific emphasis on the biological clock (*‘i-Sleep & BioClock’*). We assessed feasibility and acceptability. Pre- to post-intervention (7-week) changes in insomnia, depression, anxiety, chronotype, and quality of life were evaluated. Of 101 included students, 81 accessed the platform, 41 initiated the intervention, and 13 completed the intervention. Post-test response was 39%. Students rated the intervention with good acceptability and the platform with excellent usability. Study completers (*n* = 39) showed large improvements in insomnia severity (*p* < 0.001, *d* = 1.04), and moderate improvements in depression (*p* < 0.001, *d* = 0.63), anxiety (*p* < 0.001, *d* = 0.35), and functioning (*p* < 0.001, *d* = 0.56). No significant changes were found in quality of life (*p* = 0.07, *d* = 0.19). To summarize, the intervention had low uptake but moderate adherence and fairly good acceptability along with important improvements in sleep, mental health, and functioning.

**Trial registration:** ClinicalTrials.gov: NCT05363995.

## Introduction

Poor sleep and mental health problems are very common among university students, and prevalence numbers indicate a growing problem^1^. A large Norwegian study showed that the prevalence of insomnia symptoms in university students (*n* = 50,054) increased from 22.6% in 2010 to 30.5% in 2018^2^. In a study conducted in Germany and Luxembourg, sleep quality was impaired in 42.8% of the student sample, and 17.9% showed clinically relevant scores^3^. Furthermore, the National Institute for Public Health and Environment (RIVM) of the Netherlands confirmed the high prevalence of sleep disturbances in Dutch university students (*n* = 28,442) by reporting that 41% of students reported moderate to severe sleep problems^4^.

The university period in which students are transitioning from adolescence to adulthood is the peak time for the onset of the majority of common and severe mental disorders^5^. Students are exposed to many potential stressors during this period, such as moving abroad, financial difficulties, and high academic demands^6^. Mood- and anxiety disorders, as well as addictive behaviour and substance use disorders, are most likely to emerge at this age^5^. The relationship between sleep and mental health problems is known to be bi-directional; insomnia can contribute to the onset of psychological disorder but psychological disorders can also lead to sleep problems. Consequently, preventing and treating sleep problems in university students is crucial for the prevention of mental health disease burden.

Cognitive-behavioural therapy for insomnia (CBT-I) is the preferred insomnia treatment^7^. CBT-I aims to change thoughts and behaviours interfering with sleep. While it is typically delivered face-to-face, digital options are increasingly available. Digital CBT-I self-help interventions for insomnia are considered an easily accessible and scalable first-step treatment approach^8^, which is cost-effective compared to care as usual and sleep medication^9,10^. They are effective in reducing insomnia in the adult population^11,12^. Digital CBT-I has also been shown to improve sleep and mental health outcomes in university students, with moderate to large effect sizes^13–15^. Colleges and universities have recognized the benefits of e-health interventions and initiatives, such as the Caring Universities Consortium, and are implementing these services for students^16^.

The lifestyle of university students is prone to irregular sleep–wake cycles due to varying daily schedules and demands. Additionally, students who tend to have a late chronotype—characterized by a preference for going to bed late and waking up late—also have an increased risk of experiencing social jetlag—a mismatch between their sleep schedule on study days and on weekends or free days^17^. Social jetlag can occur when the natural sleep preference conflicts with actual schedules on study days. For instance, a student with a natural sleep preference of 2:00 a.m. to 10:00 a.m. may be required to wake at 7:00 a.m. for weekday classes, which leads to an accumulation of sleep debt during the week, followed by attempts to ‘catch up’ on sleep during the weekend. This, in combination with late chronotype and persistent instability in sleep patterns, can contribute to increased internal desynchrony, resulting in the misalignment of the body’s internal clocks^18,19^. These disruptions of the circadian system can significantly impact the health and wellbeing of students^19^. Chronobiological additions to CBT-I, such as timed light exposure, have recently been evaluated, showing a significant improvement on insomnia symptoms and clinically relevant effects in the long-term^20^. This is of particular importance for the student population, who tend to struggle with establishing and maintaining healthy routines.

Therefore, we extended a digital CBT-I intervention (*‘i-Sleep’*) by adding elements to target the biological clock. More emphasis was placed on topics such as circadian rhythm disruption, light exposure, chronotype, social jetlag, and other issues related to the biological clock. In addition, the intervention was tailored to the student population. The extended version was named *‘i-Sleep & BioClock’*. Due to the new intervention elements and new target group of this intervention, we conducted an open pilot study to evaluate the intervention content in the student population and to help identify any potential challenges prior to a larger-scale effectiveness trial.

The primary aim of this study was to examine different aspects of feasibility and acceptability of the *‘i-Sleep & BioClock’* intervention. Secondly, we aimed to investigate the preliminary pre-post effectiveness on sleep, depression, anxiety, functioning, and quality of life in university students. We hypothesized that the intervention would be feasible and acceptable to students and that there would be significant improvements in pre- to post-outcomes of insomnia and mental health symptoms.

## Methods

### Study design

The study had a pre-test post-test design. The study was conducted as a part of the Caring Universities Consortium^16^, the Dutch branch of the World Health Organization World Mental Health International College Student (WMH-ICS) initiative^21^. The CONSORT-EHEALTH checklist was used for this study^22^. The Scientific and Ethical Review Board (VCWE) of the Faculty of Behaviour & Movement Sciences, VU University Amsterdam approved all procedures. The study was in line with the standards set by the Declaration of Helsinki and was performed in accordance with the ethical regulations and practices stipulated by the ethics committee. The study was registered at Clinicaltrials.gov (NCT05363995).

### Eligibility criteria

Inclusion criteria were (1) being a Bachelor, Master, or PhD student affiliated with one of the participating universities (Leiden University, VU Amsterdam, University of Utrecht, Maastricht University, Erasmus University Rotterdam, Universiteit van Amsterdam, and Inholland University of Applied Sciences), (2) being fluent in English or Dutch Language, (3) being 16 years or older, (4) having at least sub-threshold insomnia severity (Insomnia Severity Index; ISI ≥ 8), and (5) being willing to participate in the e-health intervention.

The exclusion criteria were (1) not giving informed consent, (2) not completing the baseline assessment, (3) scoring < 8 on the ISI, and (4) current risk for suicide. Students were asked whether they had suicidal thoughts in the past 12 months. After positive response, they were asked “About how many months in the past 12 months did you think about how you might kill yourself or work out a plan of how to kill yourself?” and “In the next 12 months, what is the likelihood that you will act on those thoughts of killing yourself?”. If the answers were having thoughts about suicide for at least 1 month in the past 12 months and if the likelihood of acting on these thoughts was anything other than “Not likely at all”, they were excluded from the study and referred to other services.

In the present study, we did not perform a formal sample size calculation as we focused on the intervention’s feasibility and acceptability. Based on previous similar studies^23^, we intended to include a minimum of 50 participants to examine our main study objectives.

### Intervention

*‘i-Sleep & BioClock’* is a guided digital self-help program, which is based on cognitive behavioural therapy for insomnia. *‘i-Sleep & BioClock’* is based on the *‘i-Sleep’* intervention^24^, an established CBT-I intervention^25^. We adapted the program by tailoring it to meet the specific needs of the university students by including more interactive and engaging features, re-writing case examples to fit the student lifestyle, and providing student-specific information, such as the importance of sleep for studying and creativity, the effects of pulling all-nighters before exams or deadlines, and adjusting the sleep advice taking into account the different and possibly sleep-disrupting living situations (e.g. student housing). Furthermore, we extended the intervention by adding elements of the biological clock, such as information about light exposure and social jetlag, and a chronotype assessment with the Morningness-Eveningness-Questionnaire with feedback in the first module^26^.

The *‘i-Sleep & BioClock’* intervention is delivered in a guided digital format. It consists of one introductory module followed by five main modules. Participants were recommended to finish one module per week. Each module took between 30 and 60 min to complete and included homework. In addition, students are asked to keep a record of their sleep and light exposure habits in the form of an online diary. Students received automated graphs based on their diary input to monitor their personal progress. The *‘i-Sleep & BioClock’* intervention was designed to target both insomnia and circadian rhythm disturbances. To target insomnia, the intervention included components focused on improving sleep-related lifestyle habits, reducing hyperarousal through relaxation techniques, and addressing dysfunctional beliefs about sleep through cognitive restructuring. In parallel, the intervention addressed circadian misalignment by promoting regular sleep–wake schedules (reducing social jetlag), educating about chronotypes (chronotype assessment using the Morningness–Eveningness Questionnaire, with personalized feedback on how students could adapt their routines to better align with their biological clock) and encouraging adequate light exposure, especially in the morning (at least 15–30 min bright light/time spent outside before noon). The following topics were covered per module: (introductory module) goal setting and assessment of sleep and light exposure using the diary during a 7-day baseline period in which the main modules were locked, (module 1) psychoeducation on sleep and the biological clock, sleep hygiene, and lifestyle assessment, (module 2) stimulus control and sleep restriction, (module 3) worrying and relaxation exercises, (module 4) changing dysfunctional cognitions about sleep, and (module 5) summary module and plan for the future.

The intervention was guided by e-coaches, who were Master’s students of psychology under the supervision of a licensed therapist. The coaches performed a short intake call to establish a personal connection with the coachee, and to increase motivation and commitment to the program. Furthermore, e-coaches provided asynchronous online textual feedback on the exercises and progress of the participants after each completed module within three working days via a chat on the online platform. The e-coach had access to participants’ completed exercises and provided personalized feedback, which focused on correcting misunderstandings, offering additional information when needed, and motivating students to stay engaged with the intervention.

### Procedure

Students were recruited from May 1st, 2022, until December 1st, 2022, via university staff (student counselors, student psychologists, and study advisors), social media, on-site advertisements (workshops and courses at the participating universities), and the annual Caring Universities mental health screening survey^27^. The survey assesses sleep and mental health problems, providing detailed feedback and recommendations to start an appropriate e-health intervention. Students who were interested in participating were directed to the Caring Universities platform, where they read the information letter and signed digital informed consent before completing the online questionnaires (pre-test). Students chose an e-coach and received log-in credentials to the platform. In the introductory module, students were instructed to fill in the pre-assessment sleep diary for research purposes for seven days before starting module 1 of the intervention. Module 1 was locked during these seven days. After seven weeks, they received the same set of questionnaires (post-test) and were instructed to fill in the sleep diary for seven days.

### Measures

All measures were self-reported and administered online. Sleep and mental health outcomes were measured at pre-test (T0) and post-test after seven weeks (T1). Acceptability outcomes were measured after completion of each module and at post-test (T1). Sleep and light exposure outcomes were measured throughout the study.

### Feasibility and acceptability

We examined several aspects of feasibility and acceptability.

#### Intervention uptake

We examined the proportion of students who initiated the main modules of the intervention out of all included participants.

#### User activity

User activity data was analysed with the number of times logged in and the average time spent on the platform.

#### Adherence

We assessed the number of modules completed in students who initiated the intervention, further referred to as module completion rate. A module is defined as completed if a participant has closed the final page of the module. We also assessed the number of intervention completers, further referred to as intervention completion rate. Participants were considered intervention completers if they followed at least four out of the five main modules, as the fifth module is a summary module and does not contain new information. Questionnaires assessing reasons for intervention dropout were sent to participants if they were inactive on the platform for 3 weeks.

#### Module evaluations

After each module, participants rated its usefulness on a scale of 0 (*not useful at all*) to 10 (*very much useful*), whether its goals and content were clear, whether it was easy to navigate, and whether it was appropriate in length. Furthermore, participants were asked to provide written feedback on what they liked and disliked about the module and their opinions on what should be improved.

#### Acceptability of the intervention

Acceptability of *‘i-Sleep & BioClock’* was measured using the Client-Satisfaction-Scale (CSQ-8)^28^ at post-test. It consisted of eight items rated on a 4-point scale. Total scores are ranging from 8 to 32, with higher scores indicating greater acceptability and satisfaction with the program. The cut-off for acceptable satisfaction was 20 points, representing the median score.

#### Acceptability of the platform

The platform’s acceptance was examined with the System-Usability Scale (SUS-10)^29^ at post-test. The items are scored on a 5-point Likert Scale. Total scores are scaled to a range of 0–100, with scores of 85 or higher representing exceptional usability and scores below 70 representing unacceptable usability^30^.

#### Acceptability of the sleep and light exposure diary

We asked participants at post-test which device they used to keep the diary and how they would rate its usefulness. We furthermore asked them whether the sleep diary was usable, easy to understand, and easy to navigate.

#### Therapeutic alliance

Satisfaction with the e-coaches and therapeutic alliance was measured with the Working Alliance for guided Internet Intervention Scale (WAI-I)^31^ at post-test. The scale consists of 12 items rated on a 5-point Likert scale. Scores range from 12 to 60, with higher scores indicating better therapeutic alliance.

### Sleep outcomes

#### Insomnia severity

The Insomnia Severity Index (ISI) was used to assess the severity of insomnia symptoms over the past two weeks^32^. It covers problems falling asleep or maintaining sleep, early awakenings, as well as the daytime consequences of sleep problems using a 5-point Likert scale. Total scores categories are absence of insomnia (0–7); sub-threshold insomnia (8–14); moderate insomnia (15–21); and severe insomnia (22–28). The internal consistency of the ISI in our student sample was α = 0.78 in study completers at baseline.

#### Sleep diary outcomes

The diary includes the outcomes: *sleep onset latency* [SOL; minutes from ‘lights off’ until sleep onset]*, wake after sleep onset* [WASO; total minutes awake between sleep onset and final awakening]*, early morning awakening* [EMA; number of minutes between final awakening and getting up], time in bed [TIB; number of minutes between lights off and getting up], *total sleep time* [TST; TIB–SOL–WASO–EMA]*,* and *sleep efficiency* [SE; TST/TIB *100]*. Sleep quality* and *the feeling of being well-rested* in the morning were rated from 0 to 10; the higher, the better. We instructed students to fill in the sleep and light exposure diary for seven days in the first week and in the week after the T2 measurement. However, it was also recommended to fill in the diary during the intervention period, to monitor progress and to facilitate the sleep restriction method.

### Mental health outcomes

#### Depression severity

Depressive symptoms were measured with the Patient-Health-Questionnaire 9 (PHQ-9), asking about the past two weeks^33^. This questionnaire covers each of the nine DSM-IV criteria for depressive disorder, asking about the frequency of occurrence on a 4 -point scale. Scores can range from 0 to 27, with higher scores representing higher severity. The internal consistency of the PHQ-9 in our sample was α = 0.78 in study completers at baseline.

#### Anxiety severity

Anxiety symptoms were measured with the Generalized-Anxiety-Disorder Scale (GAD-7), assessing symptoms during the past two weeks on a 4-point scale^34^. It can generate a total score of 0–21, with increasing scores representing higher symptoms. The internal consistency of the GAD-7 in our sample was α = 0.90 in study completers at baseline.

#### Functioning

The Work and Social Adjustment Scale (WSAS) was used to assess impairment in functioning due to sleep problems^35^. It covers difficulties in functioning in the context of work, home management, social leisure, private leisure, and relationships and rates the five items on a 9-point scale. Scores are ranging from 0 to 40, with lower scores representing better functioning. The internal consistency of the WSAS in our sample was α = 0.81 in study completers at baseline.

#### Quality of life

The Mental Health Quality of Life Scale (MHQoL) was used to measure quality of life^36^. It covers the dimensions self-image, independence, mood, relationships, daily activities, physical health, and future, and includes a visual analogue scale of general psychological wellbeing. Scores range from 0 to 21, with higher scores indicating a better quality of life. The internal consistency of the MHQoL in our sample was α = 0.73 in study completers at baseline.

### Biological clock outcomes

#### Munich chronotype questionnaire

From the Munich Chronotype questionnaire (MCTQ), we reported three outcomes. Firstly, chronotype, referring to the mid-point of sleep on free days adjusted for sleep debt during the week. Secondly, social jetlag, which is the discrepancy in the mid-point of sleep between free days and work/study days. Thirdly, average sleep duration (time from sleep onset to final awakening), which is the weighted average of the sleep durations on work and work-free days in a week^37^.

#### Light exposure diary outcomes

In the sleep & light exposure diary, the *total time of daylight exposure* was assessed. We asked participants to report their time spent outside, without a roof above their head, each day. We adjusted the time spent outside to the total minutes of actual daylight exposure, by subtracting the time spent outside before sunrise and after sunset.

### Statistical analysis

Data analysis was carried out in R version 4.2.1. Baseline characteristics were compared between those who did not initiate the intervention (did not initiate the program or only did the introductory module, *n* = 60), those who did part of the intervention (who completed modules 1, 2 or 3, *n* = 28), and those who completed the intervention (who completed at least 4 main modules, *n* = 13). We compared baseline symptom severity of those who never logged in versus those who did, study completers versus non-completers, and intervention completers versus non-completers.

We calculated the descriptive statistics for acceptability and feasibility measures for those who initiated the main modules (further referred to as intervention initiators, *n* = 41). For the adherence rate, we calculated both the intervention completion rate, which is the percentage of participants who completed the intervention (at least 4 main modules) out of the total number of intervention initiators (started the main modules) as well as the module completion rate, which is total number of modules completed divided by the total possible numbers of modules of all intervention initiators.

Analysis of preliminary effectiveness was performed based on intention-to-treat using the data of study completers (*n* = 39) no matter how many modules they completed. Sensitivity analysis was conducted for intervention initiators. We conducted paired sample t-tests to compare pre- and post-test measures or Wilcoxon signed-rank tests in case the data were not normally distributed. We tested the dose–response relationship, with adherence (number of modules completed) as the independent variable and change in insomnia as the dependent variable, in a linear regression model. For the MCTQ variables, we used multiple imputations with predictive mean matching because of missing data in the variable workdays/week due to a technical error. Imputation was performed using the R package *mice*, and was repeated 100 times with 25 iterations^38^.

Analysis of sleep diary outcomes was performed based on intention-to-treat and with a sensitivity analysis including only intervention initiators. For sleep diary outcomes, we examined the trend over time. To make use of all available data points, these outcomes were analysed through linear mixed models, and negative binominal mixed models for skewed variables, using the R package *nlme* and *MASS*^39,40^. Random intercepts and slopes or random intercepts only were considered and models were compared using likelihood ratio test statistics. Power transformation was applied to the negatively skewed SE variable (SE/100*3) and square root transformation was used for the skewed light exposure variable. SOL, WASO, and EMA were recoded to represent counts (1 count per 15 min) to account for large numbers of values of zero. Continuous autocorrelation structure of order 1 was used to account for missing data and unequally spaced observations.

All statistical tests were checked for their assumptions. Effect sizes of within-subject changes were reported using Cohen’s *d*, with < 0.2 interpreted as a small effect, 0.5 as moderate, and > 0.8 as a large effect^41^. The significance level was set to *p* = 0.05, two-tailed.

## Results

### Participants

**Fig. 1:**
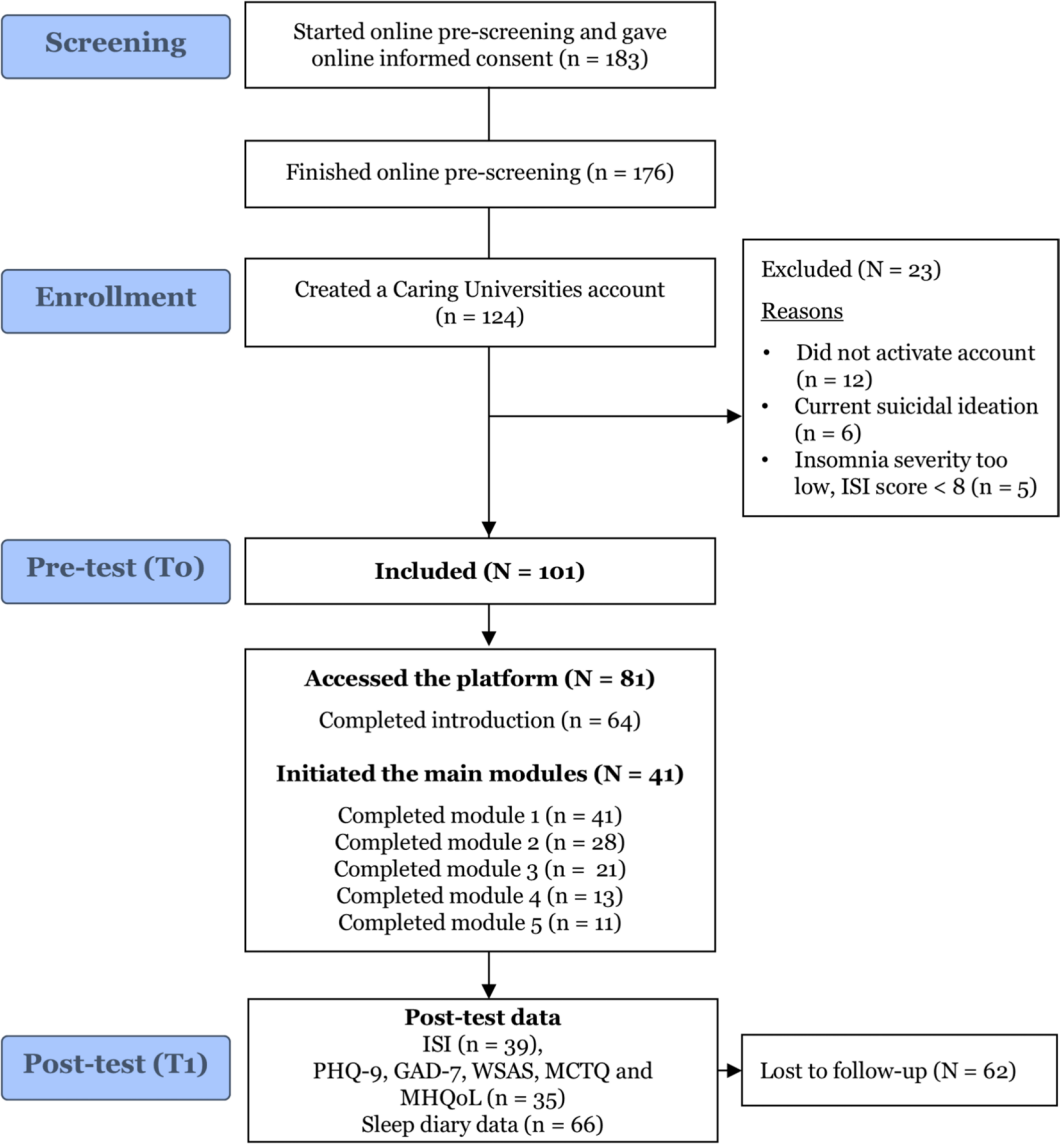
CONSORT (Consolidated Standards of Reporting Trials) flow diagram.

Initially, 183 students signed up for the study, of which 124 created an account, of which 23 were excluded. A total of 101 students were included in the study, as shown in the flow diagram Fig. 1. More than half of the sample was recruited via the Caring Universities mental health screening survey (*n* = 51). University mail and newsletters accounted for 17 participants, and student counselors for an additional ten. Table S1 of the Supplementary Material shows an overview of all recruitment methods.

Of the 101 included students, 20 never logged in to access the platform. Subsequently, only half of the 81 students who accessed the platform initiated the main modules (*n* = 41).

Table 1 shows the demographic characteristics of students who were intervention completers (*n* = 13), students who did part of the intervention (*n* = 28), and those who did not start the intervention (*n* = 60). None of the baseline characteristics were significantly different between these groups. An additional overview of the baseline characteristics of subsets of participants included in various analyses can be found in Table S2 (Supplementary Material).

**Table 1:** Demographic characteristics.

Demographic characteristics	Intervention completers (n = 13)	Did part of the intervention (n = 28)	Did not start the intervention (n = 60)	
**Age, years**				
Mean (SD)	25.3 (4.1)	25.3 (8.8)	23.5 (7.2)	*F*(2) = 0.7, *p* = 0.50
Range	19–32	16–58	17–68	
**Gender, n (%)**				
Female	8 (61.5)	18 (64.3)	45 (75.0)	*Χ*^2^ (4) = 8.0, *p* = 0.09
Male	4 (30.8)	10 (35.7)	15 (25.0)	
Other	1 (7.7)	–	–	
**University, n (%)**				
University of Amsterdam	6 (46.1)	9 (32.2)	23 (38.3)	*χ*^2^(12) = 15.2, *p* = 0.23
Leiden University	–	10 (35.7)	14 (23.3)	
Vrije Universiteit Amsterdam	2 (15.4)	6 (21.4)	13 (21.7)	
Erasmus University Rotterdam	2 (15.4)	–	4 (6.7)	
Maastricht University	1 (7.7)	1 (3.6)	3 (5.0)	
Utrecht University	2 (15.4)	2 (7.1)	1 (1.7)	
Inholland Hogeschool	–	–	2 (3.3)	
**Level of education, n (%)**				
First-year student	3 (23.1)	7 (25.0)	14 (23.3)	*χ*^2^(10) = 17.3, *p* = 0.07
Second-year student	–	2 (7.1)	13 (21.7)	
Third-year student	–	1 (3.6)	8 (13.4)	
Fourth-year student	–	3 (10.7)	2 (3.3)	
Master student	10 (76.9)	14 (50.0)	23 (38.3)	
PhD student	–	1 (3.6)	–	
**Nationality, n (%)**				
European (Dutch)	9 (69.2)	16 (57.1)	30 (50.0)	*χ*^2^(8) = 4.0, *p* = 0.86
European (not Dutch)	2 (15.4)	7 (25.0)	21 (35.0)	
Asian	2 (15.4)	4 (14.3)	6 (10.0)	
South American	–	1 (3.6)	2 (3.3)	
North American	–	–	1 (1.7)	
**Relationship status, n (%)**				
Single	8 (61.5)	17 (60.7)	38 (63.3)	*χ*^*2*^(2) = 0.1, *p* = 0.97
Other	5 (38.5)	11 (39.3)	22 (36.7)	
**Children, n (%) yes**	–	–	2 (3.3)	*χ*^*2*^(2) = 1.4, *p* = 0.50
**Part-time job, n(%) yes**	3 (23.1)	16 (57.1)	31 (51.6)	*χ*^*2*^(2) = 4.3, *p* = 0.11
**Duration of sleep problems, years**				
< 1 year	3 (23.1)	10 (35.7)	19 (31.7)	*χ*^*2*^(4) = 1.2, *p* = 0.87
1 – 4 years	5 (30.8)	10 (35.7)	20 (33.3)	
> 4 years	6 (46.1)	8 (28.6)	21 (35.0)	
**Use of medication or psychotherapy, n (%)**				
Medication	-	7 (25.0)	8 (13.3)	*χ*^*2*^(6) = 9.6, *p* = 0.14
Psychotherapy	-	6 (21.4)	9 (15.0)	
Both	1 (7.7)	1 (3.6)	4 (6.7)	
None	12 (92.3)	14 (50.0)	39 (65.0)	
**Baseline symptoms, mean (SD)**				
Insomnia Severity (ISI)	16.1 (4.0)	16.1 (4.1)	16.8 (3.9)	*F*(2, 98) = 0.4, *p* = 0.70
Depression (PHQ-9)	10.1 (4.5)	11.7 (5.2)	11.6 (4.1)	*F*(2, 98) = 0.7, *p* = 0.50
Anxiety (GAD-7)	8.7 (6.7)	5.2 (6.8)	9.3 (4.5)	*F*(2, 98) = 2.3, *p* = 0.11
Functioning (WSAS)	16.8 (6.2)	18.4 (8.7)	21.1 (7.0)	*F*(2, 98) = 2.5, *p* = 0.09
Quality of Life (MHQoL)	13.7 (3.8)	13.6 (3.0)	12.3 (2.5)	*F*(2, 98) = 2.6, *p* = 0.08

*Note*. Intervention completers = All participants who did 4 or 5 modules. Did part of the intervention = did 1, 2 or 3 modules, Did not start the intervention = did 0 modules or only the introductory module. Use of medication: any current medication use; non-specified. Use of psychotherapy: any current psychotherapy; non-specified.

### Feasibility and acceptability

#### Uptake, user activity and adherence

Among the 101 participants, 81 students (80.1%) accessed the platform and initiated the introduction module. In terms of intervention uptake, 41 (40.5% of all included students) started the main modules of intervention and completed at least module 1. Of students who initiated the intervention (*n* = 41), 13 students completed the intervention (at least 4 modules). Therefore, the intervention completion rate was 31.7%. When considering the total number of modules completed by intervention initiators, 155 out of the 246 (41*6) possible modules were completed, yielding a module completion rate of 63%. The average number of main modules completed was 2.8 modules (SD 1.6) among intervention initiators. The average number of logins was 36 (SD 23.0), with a median of 30 times, and the average time spent on the platform was 1 h and 55 min (*median* = 1 h 42 min, *max* = 6.5 h). An overview of the intervention uptake and number of modules completed per student can be found in Fig. 1.

Thirty-nine % of all 101 included participants (*n* = 39) were study completers (meaning they completed the ISI at post-test), and 62 were study non-completers (did not complete the post-test questionnaire). Of the 13 students who completed the intervention, 12 (92%) also completed the study. An overview of the number of modules completed by study completers and non-completers can be found in Figure S1 and S2 in the Supplementary Material.

#### Reasons for discontinuation

Of the 88 students who did not finish the intervention, only 14 (16%) completed the questionnaire about reasons for discontinuation. The most common reasons for intervention discontinuation were lack of time (*n* = 9, 37.5%), lack of motivation (*n* = 3, 12.5%), the intervention being too demanding (*n* = 3, 12.5%). The most demanding part of the intervention was the sleep restriction. Some students believed that the intervention was not what they really needed (*n* = 2) and some preferred face-to-face help (*n* = 2). No dropout was attributed to coach-related reasons. An overview of dropout reasons can be found in Table S3 of the Supplementary Material.

#### Module evaluations

After completion of each module, students were asked to give a module evaluation. Overall, students who completed the modules were satisfied with the respective modules. The modules were evaluated with scores ranging from 6.86 to 7.51 out of 10, as shown in Table 2. Students furthermore gave textual feedback for each module. Positive remarks were that the intervention was clear, informative, and scientifically underpinned. Students especially appreciated the interactive elements, such as videos, exercises, self-reflection questions, which made the material more engaging. The inclusion of practical tools, such as actionable tips and specific examples was seen as particularly helpful. Students furthermore value a personalized approach, with content tailored to their prior knowledge, needs, and personal situation. Negative remarks included module 1 (psychoeducation) being too extensive and overwhelming due to the number of suggested lifestyle changes. In addition, the timing of certain exercises such as sleep restriction at times conflicted with students’ schedules. More details regarding module evaluations can be found in Tables S4 and S5 of the Supplementary Material.

**Table 2:** Module evaluation in terms of general usefulness.

Module	Title	N	Usefulness score (SD)
Module 1	Sleep and your habits	41	7.51/10 (1.23)
Module 2	Improving your sleep pattern	27	7.48/10 (1.20)
Module 3	Worrying and relaxation	21	6.86/10 (2.47)
Module 4	Changing your thoughts	12	7.46/10 (1.39)
Module 5	And now?	11	6.64/10 (2.06)

*Note.* Scores 0 = *not useful at all*, 10 =* very useful*.

#### Acceptability of the i-Sleep & BioClock intervention

The CSQ-8 was filled in by 24 of the 41 intervention initiators. Their mean score was 23.38 (SD 2.9), with a range of 18 to 29. The percentage of people who found the program acceptable was 87% (*n* = 21 out of 24, scoring above the cut-off of 20 points). The quality of the program was rated with a mean score of 3.1 out of 4 points (*“good”*), and overall satisfaction with a mean score of 3.1 out of 4 points (*“mostly satisfied”*).

#### Acceptability of the Caring Universities platform

In intervention initiators who SUS-10 post-test data (*n* = 24 out of 41), the SUS-10 had a mean score of 83.8 (SD 12.8) with a range of 50 to 100, which indicates very good or excellent usability of the platform^42^. More than two-thirds of the students (67%) rated the system with excellent usability.

#### Acceptability of the sleep and light exposure diary

Of all 101 participants, 66 used the sleep and light exposure diary at least once. The average number of entries per participant was 20 nights (*median* = 11, *maximum* = 99). Of the 27 post-test responders, most indicated using the diary on their mobile phones (*n* = 17, 63.0%), some on their computer or laptop (*n* = 8, 29.6%), or on tablet/iPad (*n* = 2, 7.4%). Students (*n* = 28) rated the usefulness of the diary with 7.2 (SD 2.1) out of 10 (*“very much useful”*). Although many students did not fill in the diary at all, participants of the study completers rated the diary positively. Most found the explanations in the diary straightforward and the graphs easy to understand, 89% and 82%, respectively. A large majority (78%) agreed that the diary was not unnecessarily complex. However, some (36%) found that the diary took too long to complete. Detailed depiction of sleep diary items and their evaluation can be found in Table S6 and S7 of the Supplementary Material.

#### Therapeutic alliance

Among the 23 of the 41 intervention initiators who filled in the WAI-I, the mean score was 45.8 (SD 6.3) which is 70% of the highest achievable value.

### Preliminary effectiveness

#### Insomnia severity and mental health outcomes

Paired samples t-tests of study completers (*n* = 39) showed a significant reduction of insomnia severity from pre-test to post-test, with a mean difference of − 4.69 (SD 5.19) on the ISI, *t*(38) = 5.65, *p* < 0.001, indicating a large effect size *d* = 1.04, as shown in Table 3. A sensitivity analysis among intervention initiators shows a very large effect size (*d* = 1.34) in insomnia severity for those who initiated the main modules (*n* = 27). Details for the sensitivity analysis for questionnaire outcomes can be found in the Supplementary Material Table S8. The number of students whose insomnia can be defined as severe (ISI score of ≥ 15) decreased from 59% at pre-test (*n* = 23) to 28% at post-test (*n* = 11). Figure S3 illustrates the distribution of responses for each item of the ISI in all included participants (*n* = 101).

**Table 3:** Pre-test post-test differences of insomnia severity and mental health outcome measures.

Measure	N	Pre-test, mean (SD)	Post-test, mean (SD)	*p* value	Cohen’s *d *(95% CI)
ISI insomnia severity	39	15.49 (3.80)	10.79 (5.09)*	**< 0.001**	1.04 (0.57, 1.51)
PHQ-9 depression	35	11.34 (4.79)	8.31 (4.89)*	**< 0.001**	0.63 (0.14, 1.10)
GAD-7 anxiety	35	8.31 (5.98)	6.40 (4.86)*	**0.009**	0.35 (− 0.12, 0.82)
WSAS functioning	35	19.29 (7.85)	14.63 (8.76)*	**0.003**	0.56 (0.08, 1.04)
MHQoL quality of life	35	12.71 (3.41)	13.34 (3.04)	0.067	− 0.19 (− 0.66, 0.28)
MHQoL overall mental wellbeing	35	5.43 (1.99)	5.94 (1.68)	0.059	− 0.28 (− 0.75, 0.19)
MCTQ chronotype (mid-sleep, local time)	14	04:41 (02:10)	05:03 (01:18)	0.508	− 0.20 (− 0.94, 0.55)
MCTQ average sleep duration	33	7 h 27 min (1 h 18 min)	8 h 14 min (64 min)*	**0.044**	− 0.52 (− 1.00, − 0.04)
MCTQ absolute social jetlag	33	58 min (46 min)	1 h 07 min (49 min)	0.318	− 0.19 (− 0.66, 0.28)

*Note. ISI* Insomnia Severity Index: 7-item insomnia scale; scores ranging from 0 to 28; higher scores indicate higher insomnia severity. *PHQ-9* 9-item Patient Health Questionnaire; scores ranging from 0 to 27; higher scores indicate higher depression severity. *GAD-7* 7-item Generalized Anxiety Disorder scale; scores ranging from 0 to 21; higher scores indicate higher anxiety severity. *WSAS* 5-item Work and Social Adjustment scale; scores ranging from 0 to 40; higher scores indicate higher impairment in functioning. *MHQoL* 8-item Mental Health Quality of Life Questionnaire; scores ranging from 0 to 21; higher scores indicate better quality of life. *MHQoL* Overall mental wellbeing rated on a scale of 1 (*worst possible wellbeing*) to 10 (*best possible wellbeing*). *MCTQ* 17-item Munich Chronotype Questionnaire; self-report sleep measure comparing sleep outcomes on work/study days versus free days. *h* hours, *min* minutes, *MCTQ* chronotype in local time.

*p < 0.05, statistically significant difference to pre-test.

Depression and anxiety symptoms also decreased from pre- to post-test, depression with *M* = − 3.03 (SD 3.44), *t*(34) = 5.2, *p* < 0.001, and anxiety with *M* = − 1.91 (SD 3.58), *Z* = 427.5, *p* < 0.05. Impairment in functioning was furthermore significantly reduced by 4.66 (SD 8.62), *t*(34) = 3.20, *p* < 0.05. Effects on quality of life, *t*(34) = 1.89, *p* = 0.07, and overall mental wellbeing were not significant *t*(34) = 1.95, *p* = 0.06.

#### Chronotype and light exposure

The average chronotype (*n* = 14) measured by the MCTQ shifted from 4:41 h to 05:03 h, which is a non-significant difference towards more eveningness, *t*(13) = − 0.68, *p* = 0.51. This means that the mid-point of sleep got delayed from pre-test to post-test by 22 min. Average social jetlag showed a non-significant increase by 9 min, *Z* = 212, *p* = 0.32. This means that the discrepancy in the mid-point of sleep between work/study days and free days increased slightly. Average sleep duration based on MCTQ increased by 47 min, *t*(32) = 2.09, *p* = 0.04. Among study completers (*n* = 39), average time attempting to sleep shifted earlier both on work/study days (from 00:26 h at baseline to 23:59 h at post-test) and free days (from 00:52 h to 00:33 h). Wake times remained relatively stable, shifting slightly later on both workdays (from 08:15 h to 08:25 h) and free days (from 09:38 h to 09:51 h). This suggest that the observed increase in total sleep time was more driven by earlier bedtimes rather than later wake times. The changes in sleep and wake times were not statistically significant.

Total daylight exposure measured with the light exposure diary was, on average, 45 min per day at the start of the study, and there was a very slight but significant increase in light exposure over time (*B* = 0.0001, SE = 0.006, *p* = 0.03).

#### Dose–response relationship

Adherence to the program influenced the change in insomnia symptoms. The overall linear regression model showed that the number of modules completed was a significant predictor of change in the ISI, *t*(37) = − 2.4, *p* = 0.02. The beta coefficient of *B* = − 0.87 (SE = 0.36) showed that with each completed module, the decrease in insomnia symptoms became more prominent.

#### Sleep diary outcomes

Sixty-six participants filled in the sleep diary at least once. The mean number of entries was 20 nights (*median* = 11, *max* = 99). Trends of sleep diary outcomes were analyzed over time with intention to treat approach using all available data. At the beginning of the intervention period, the average total sleep time was estimated to be 7 h and 17 min and there was a significant increase over time. Each day in the study period was associated with a 0.32-min increase in total sleep time (SE = 0.12, *p* = 0.009) meaning there was an average increase in total sleep time of 16 min across the 7-week study period. Sleep efficiency also increased over time. For each additional day, the estimated change in sleep efficiency (%) was approximately 0.11% (SE = 0.07, *p* < 0.001), meaning an average increase of 5.39% across the study period. Sleep onset latency, wake after sleep onset, and early morning awakening showed a statistically significant decrease over time, with (*B* = 1.00, 95% CI 0.99, 1.00, *Z* = − 2.49, *p* = 0.013), (*B* = 0.99, 95% CI 0.99, 1.00, *Z* = − 2.63, *p* < 0.001), and (*B* = 0.99, 95% CI 0.99, 1.00, Z = − 5.50, *p* < 0.001), respectively. This implies an estimated overall decrease of 15.7% in sleep onset latency, a decrease of 23.7% in wake after sleep onset, and a decrease of 36.7% in early morning awakening over the 7-week study period. Furthermore, sleep quality and having a refreshed feeling in the morning both increased over time with *B* = 0.01 (SE = 0.003, *p* < 0.001). This means that the sleep quality increased on average from a score of 5.50 to 5.99 (0 = *worst possible*, 10 = *best possible*) across the 7-week period. Having a refreshed feeling in the morning increased from 4.94 to 5.43 (0 = *worst possible*, 10 = *best possible*). Details for the sensitivity analysis in intervention initiators (n = 41) can be found in the Supplementary Material Table S9.

## Discussion

This study explored the feasibility, acceptability, and preliminary effectiveness of a sleep and biological clock intervention for university students, the *‘i-Sleep & BioClock’* intervention. In terms of feasibility, we investigated whether an established CBT-I intervention can be used for a different target population, and assessed the potential to successfully integrate the intervention into an existing infrastructure. Regarding acceptability, we evaluated how relevant and adequate the intervention content is to university students, and we identified necessary modifications to the intervention content before executing a larger effectiveness trial.

Intervention initiators indicated good acceptability of the intervention and excellent usability of the platform. Overall, the intervention was perceived positively, as indicated by module evaluations and usefulness ratings. Ratings of the CSQ-8 were high, which is in line with similar digital interventions^43,44^. Platform usability ratings were also comparable to other interventions^43,45^. The students’ feedback on the intervention content was positive, clear, informative, and scientifically underpinned. Students rated Module 1 (psychoeducation), Module 2 (sleep restriction), and Module 4 (cognitive restructuring) as the most useful modules of the intervention. On the other hand, some students indicated that the sleep restriction method was perceived as invasive, especially during exam periods or internships. Daily sleep monitoring through logging in via the online diary was perceived as burdensome by some students. While the majority of students agreed that the sleep and light exposure diary was not unnecessarily complex, over a third reported that it took too long to complete, suggesting that time investment, rather than complexity, may have contributed to adherence challenges. In addition, time investment was the most reported reason for discontinuation with the intervention, followed by the perceived high demands of the intervention, although only few students responded to the dropout questionnaire.

In contrast to our positive findings on the acceptability of the platform and the intervention, feasibility measures revealed some issues. The intervention uptake was 40.5%, which is comparable to another digital intervention on the same platform, where 41.2% discontinued the program after the first module^43^. The low uptake is most likely related to the Caring Universities platform infrastructure, with very low-threshold access to the intervention, lack of guidance in the onboarding process, and participation in research as a prerequisite for using the intervention. This is not considered highly problematic, as early dropout leads to only a minimal loss of time and resources for both participants and researchers. The ongoing use of the program was somewhat lower than expected, but there seemed to be much variability in platform usage as reflected in variations in time spent on the platform and the number of logins. The module completion rate was 63%, and the intervention completion rate was 31.7%. This is comparable to a similar pilot study investigating a stress management intervention in students using the same online platform which had an intervention completion rate of 33.6%^43^, suggesting that disengagement with the intervention might not be related to the intervention content or delivery itself but rather connected to other factors such as personal motivation or incongruence between intervention content and personal goals. Modest adherence is common in digital interventions, especially in university students^46–48^, though, in contrast to other large-scale studies on digital CBT-I in adults with insomnia wherein a completion rate of approximately 50% or higher has been observed^49,50^. The observed adherence to the program might pose an issue, although it has been shown that module completion is not necessarily directly related to treatment outcome^51^. However, in the case of this pilot study, the number of completed modules was a significant predictor of reduction in insomnia severity from pre-test to post-test. Students who completed the intervention were also motivated to complete the study by filling in the post-test questionnaire. For students who only used part of the intervention (*n* = 28) only half also completed the study. This possibly indicates that while students are eager to utilize the program, they might not be equally motivated to engage in research. Additionally, the automated enrollment process and online nature of the study allowed students to easily withdraw from the study, contributing to the dropout rate. Nevertheless, study completers experienced significant improvements in sleep and mental health from pre-test to post-test.

Our student sample (*n* = 101) had an average insomnia severity score of 16.5 (SD 4.0) at baseline, and in intervention initiators (*n* = 41), the average was 16.1 (SD 4.1), which can be categorized as clinical insomnia with moderate severity^32^. Additionally, one-third of all students claimed to have had sleep problems for more than 4 years already. The majority of students were very dissatisfied with their sleep and indicated a severe to very severe with their daily functioning, as indicated by scores on separate items of the ISI (Fig. S3). In contrast, sleep diary data showed that the average total sleep time at the start of the intervention was already fairly long with 7 h and 17 min whereas total sleep time in other insomnia populations is often between 5 and 7 h^25,52,53^. One possible explanation for this discrepancy could be that students tend to fill in the diary on days following better nights of sleep, leading to an overestimation of their typical sleep duration. It also could be explained by unrealistic expectations of students believing they need at least 8 h of sleep and, therefore, are dissatisfied about anything less. It is also possible that students experience sleep state misperception, characterized by poor perceived sleep quality, daytime dysfunction or daytime sleepiness despite reporting normal total sleep time and sleep efficiency^54^. Another explanation could be that students might struggle with specific sleep complaints, such as irregular sleep–wake patterns, feelings of unrefreshing sleep, or trouble getting out of bed in the morning, rather than the more typical insomnia symptoms, such as falling asleep or staying asleep. These complaints may negatively impact their well-being and contribute to sleep dissatisfaction despite adequate total sleep time.

On average, study completers experienced large improvements in insomnia symptoms, and moderate improvements in anxiety, depressive symptoms, and functioning, but no significant difference in quality of life and psychological wellbeing. These pre-post findings are consistent with previous studies on internet-delivered CBT-I interventions aimed at university students, showing moderate to large effect sizes. For instance, Freeman et al. conducted a large randomized controlled trial (RCT) in 26 universities in the UK, administering the 6-session Sleepio intervention compared to usual care, and found that there were large reductions in insomnia (*d* = 1.11) after 10 weeks^48^. Similarly, Morris et al. found significant improvements in sleep quality with a large within-group effect size of *d* = 1.04 for participants who followed a 6-week CBT-I intervention^55^. A somewhat lower effect of the Sleepio intervention on insomnia was found in a trial by Denis et al. aimed at female students with sub-threshold insomnia *d* = 0.42^56^. An overall moderate effect was also found in a meta-analysis summarizing digital and face-to-face single- and multi-component sleep interventions by Chandler et al. (*SMD* = − 0.55)^15^, and in a meta-analysis summarizing effects of psychological interventions aimed at sleep by Saruhanjan et al. *g* = 0.61^13^. In terms of secondary outcomes, other studies reported comparable or slightly lower effects of CBT-I on anxiety and depression outcomes^15,57^, and similar effects on functioning^48^, but slightly higher effects on quality of life^58^. The low effects on quality of life and psychological wellbeing might be attributable to the multifaceted nature of these concepts. Improvements in sleep might correlate with improvements in certain aspects of wellbeing, such as mood and energy levels, more than other aspects such as physical wellbeing or sense of purpose. Lastly, sleep diary outcomes showed positive trends over time and all outcomes developed in the desired direction.

While existing research supports the effectiveness of digital CBT in student populations, our study introduces the focus on regulating the biological clock as an innovative element of the intervention, by integrating themes such as chronotypes and light exposure. In addition to addressing the sleep problems, we also aimed to address the underlying circadian misalignment that is common in students. Our results showed that despite clear instructions on regularity of sleep patterns, there were no significant improvements in social jetlag from pre- to post-test, and despite instructions for more light exposure, this outcome marginally improved during the study period. Regarding sleep patterns measured with the MCTQ, wake times remained relatively stable and although we observed a trend toward earlier bedtimes, changes in sleep timing were not statistically significant. Participants entered the study with relatively high total sleep time and sleep efficiency. In such cases, consistent with CBT-I principles, improvements may manifest through earlier bedtimes since wake-up times could not be further delayed—a pattern observed in our sample. The modest shift toward earlier bedtimes may also reflect students’ intention to shift their sleep pattern to an earlier time, although external factors such as academic schedules may have played a role. In addition, chronotype did not shift during the intervention, which might be attributable to its less dynamic nature. Prior literature suggests that chronotype has both trait-like and state-like qualities^59^. In the subsequent randomized controlled trial, we will test chronotype as a moderator variable. Still, knowledge and understanding about the biological clock remains an important aspect of optimizing sleep and overall health and it might help to adopt healthier lifestyle habits even if measurable changes in this study were not evident. Light exposure, a key driver of circadian entrainment, can show relatively rapid changes in response to interventions, often within days to weeks^60^. Similarly, effective interventions targeting social jetlag have also been brief (e.g. 2 weeks)^61^.

This study had several limitations. First, since there was no control group, preliminary effectiveness outcomes must be interpreted with caution as the improvement might be attributed to spontaneous improvement or regression to the mean. Secondly, the observed rates of uptake and adherence raise questions about the feasibility of the intervention. However, these findings are typical for online self-help interventions, and the potential adverse effects of limited uptake and adherence are context-dependent and may be mitigated. Thirdly, study dropout was high creating a challenge in the assessment of acceptability and effectiveness. Methodologically, imputation is not recommended with this substantial amount of missing data. Therefore, for questionnaire outcomes, only the completer analysis was reported, and we cannot rule out that study non-completers were worse off than those who completed the study. Several strategies to improve adherence and decrease study dropout will be implemented in the RCT, including more specialized training and closer supervision for e-coaches to improve participant engagement, setting realistic expectations about the time investment involved in following the intervention, and financial compensation for measurements^62^. In particular, the reported challenges with applying sleep restriction during exam periods underscore the importance of the e-coach’s role in helping students to find realistic compromises. E-coaches should be trained to be aware of these common challenges and to support students in adapting the intervention to their personal situation, including setting manageable goals and balancing sleep recommendations with study demands. Another limitation is that we did not include qualitative evaluations such as interviews, which could have given more detailed insights and information on improvement suggestions. However, module evaluations in the form of textual feedback were used to improve the intervention and tailor it to the students’ needs. Furthermore, in this study, we conceptualized feasibility and acceptability with a specific set of parameters. However, we did not cover other relevant aspects, such as cost-effectiveness. Despite these limitations, this feasibility study has given valuable insight into the feasibility and acceptability of the intervention as well as the online platform.

Future research could investigate how CBT-I enhanced with chronobiological elements can be further optimized for student populations. This includes exploring personalization approaches based on chronotype assessments, developing adaptive intervention components (e.g., more flexible sleep restriction protocols) to accommodate demanding academic periods, and identifying additional strategies to improve adherence in this target group.To summarize, the *‘i-Sleep & BioClock’* intervention was perceived as acceptable by the student sample and could potentially be effective in improving insomnia and mental wellbeing. Although the study had a high dropout rate, the findings of this open pilot study are informative for the upcoming RCT to investigate the effectiveness of i-Sleep & BioClock compared to online psychoeducation in students.

## Supplementary Information

Supplementary Information.

## Data Availability

The datasets generated and analysed during the current study are available from the corresponding author on reasonable request, and in the DataVerse repository after study completion.
