# Supplementary material for: Open pilot study of a guided digital self-help intervention targeting sleep and the biological clock in university students using a pre-test post-test design
Source: Sci Rep. 2025 Jul 1;15:21837. doi: 10.1038/s41598-025-04891-8 (PMC12218973; doi:10.1038/s41598-025-04891-8)
Supplement: Supplementary file 1 — Supplementary Information. [file 41598_2025_4891_MOESM1_ESM.docx]

**Supplementary Material**

**Title**

Open Pilot Study of a Guided Digital Self-Help Intervention Targeting Sleep and the Biological Clock in University Students Using a Pre-Test Post-Test Design

**Authors**

Laura M. Pape, MSc.^1^, Niki Antypa, Dr. ^1^, Philip Spinhoven, Prof. dr.^1^, Annemieke van Straten, Prof. dr.^2, 3^, Sascha Y. Struijs, Dr. ^2^, Caring Universities Consortium*

**Affiliations**

^1^ *Department of Clinical Psychology, Leiden University, The Netherlands* ^2^ *Department of Clinical, Neuro- and Developmental Psychology, VU University Amsterdam, The Netherlands*
^3^ *Amsterdam Public Health Research Institute, Vrije Universiteit Amsterdam, the Netherlands*

**Corresponding author**

Niki Antypa (**[nantypa@fsw.leidenuniv.nl](mailto:nantypa@fsw.leidenuniv.nl)**)

**Table S1:** Recruitment strategies

| **How did you learn about the program? (N= 101)** | **n** |  |
| --- | --- | --- |
| Via the Caring Universities survey | 51 | |
| Via University mail/newsletter | 17 | |
| Via student counseling | 10 | |
| Via Social Media | 8 | |
| Via a fellow student | 6 | |
| Via a workshop | 3 | |
| Via the UniLife app | 2 | |
| Via Local advertisement | 2 | |
| Via a course | 1 | |
| Via University website | 1 |  |

**Table S2:** Demographic characteristics

| **Demographic characteristics** | | **All students**  **(n = 101)** | **Subset Study Completers**  **(n = 39)** | **Subset**  **Sleep Diary Analysis**  **(n = 66)** |
| --- | --- | --- | --- | --- |
|  |  |  |  |  |
| **Age, years** | |  |  |  |
|  | Mean (SD) | 24.26 (7.4) | 25.26 (7.7) | 24.3 (6.6) |
|  | Range | 16 - 68 | 18 - 58 | 16 - 58 |
| **Gender, n (%)** | |  |  |  |
|  | Female | 71 (70.3) | 28 (71.8) | 45 (68.2) |
|  | Male | 29 (28.7) | 10 (25.6) | 20 (30.3) |
|  | Other | 1 (1.0) | 1 (2.5) | 1 (1.5) |
| **University, n (%)** | |  |  |  |
|  | University of Amsterdam | 38 (37.6) | 16 (41.0) | 24 (36.4) |
|  | Leiden University | 24 (23.7) | 7 (17.9) | 17 (25.8) |
|  | Vrije Universiteit Amsterdam | 21 (20.8) | 3 (7.7) | 12 (18.2) |
|  | Erasmus University Rotterdam | 6 (5.9) | 5 (12.8) | 4 (6.1) |
|  | Maastricht University | 5 (5.0) | 2 (5.1) | 2 (3.0) |
|  | Utrecht University | 5 (5.0) | 5 (12.8) | 5 (7.6) |
|  | Inholland Hogeschool | 2 (2.0) | 1 (2.7) | 2 (3.0) |
|  |  |  |  |  |
|  |  |  |  |  |
|  | *Table continued* |  |  |  |
|  | | **All students**  **(n = 101)** | **Subset Study Completers**  **(n = 39)** | **Subset  Sleep Diary Analysis**  **(n = 66)** |
| **Level of education, n (%)** | |  |  |  |
|  | First year student | 24 (23.8) | 9 (23.1) | 18 (27.3) |
|  | Second year student | 15 (14.8) | 4 (10.3) | 6 (9.1) |
|  | Third year student | 9 (8.9) | 1 (2.7) | 3 (4.5) |
|  | Fourth year student | 5 (5.0) | 3 (7.7) | 5 (7.6) |
|  | Master student | 47 (46.5) | 21 (53.8) | 33 (50.0) |
|  | PhD student | 1 (1.0) | 1 (2.7) | 1 (1.5) |
| **Nationality, n (%)** | |  |  |  |
|  | European (Dutch) | 55 (54.4) | 24 (61.5) | 38 (57.6) |
|  | European (not Dutch) | 30 (29.7) | 11 (28.2) | 17 (25.8) |
|  | Asian | 12 (11.9) | 2 (5.1) | 9 (13.6) |
|  | South American | 3 (3.0) | 2 (5.1) | 1 (1.5) |
|  | North American | 1 (1.0) | 0 | 1 (1.5) |
| **Relationship status, n (%)** | |  |  |  |
|  | Single | 63 (62.4) | 22 (56.4) | 42 (63.6) |
|  | Other | 38 (37.6) | 17 (43.6) | 24 (36.4) |
| **Children, n (%) yes** | | 2 (2.0) | 0 | 0 |
| **Part-time job, n (%) yes** | | 50 (49.5) | 17 (43.6) | 32 (48.5) |
| **Duration of sleep problems, years** | |  |  |  |
|  | < 1 year | 32 (31.7) | 13 (33.3) | 20 (30.3) |
|  | 1 – 4 years | 34 (33.6) | 9 (23.1) | 23 (34.9) |
|  | > 4 years | 35 (34.7) | 17 (43.6) | 23 (34.8) |
| **Use of medication or psychotherapy, n (%)** | |  |  |  |
|  | Medication | 15 (14.9) | 7 (17.9) | 9 (13.6) |
|  | Psychotherapy | 15 (14.9) | 6 (15.4) | 10 (15.1) |
|  | Both | 6 (5.9) | 2 (5.3) | 2 (3.0) |
|  | None | 65 (64.3) | 24 (61.5) | 45 (68.2) |
|  |  |  |  |  |

*Note.* Subset Study Completers = All participants included in the analysis of questionnaire data. Subset Sleep Diary Analysis = All participants included in the analysis of sleep diary data. Use of medication: any current medication use; non-specified. Use of psychotherapy: any current psychotherapy; non-specified.

**Figure S1:** Adherence in study non-completers

**
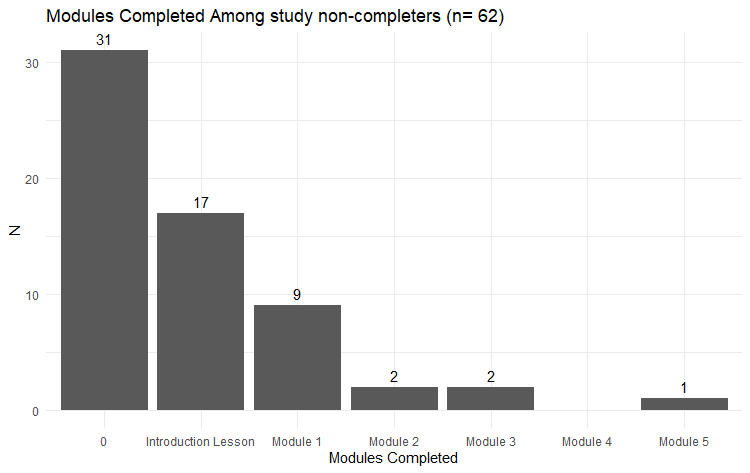
**

**Figure S2:** Adherence in study completers

**
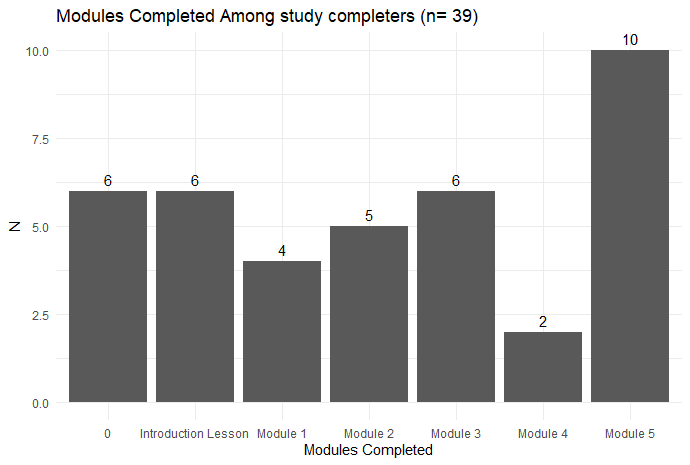
**

**Table S3:** Reasons for Dropout

| **Dropout Reasons (N= 14)** | **n** | **%** |
| --- | --- | --- |
|  |  |  |
| **Personal reasons** |  |  |
| No time | 9 | 37.5 |
| Lost interest/ Motivation | 3 | 12.5 |
| Symptoms improved | 1 | 4.2 |
| Symptoms did not improve | ⁻ | ⁻ |
| Stressful life event | 1 | 4.2 |
| Found other source of help | 1 | 4.2 |
|  |  |  |
| **Intervention-related** |  |  |
| Intervention is boring | 1 | 4.2 |
| Technical difficulties | ⁻ | ⁻ |
| Platform difficult to navigate | 1 | 4.2 |
| Intervention too demanding | 3 | 12.5 |
| Intervention too complicated | ⁻ | ⁻ |
| Not what I need | 2 | 8.3 |
| Problems with internet connection | ⁻ | ⁻ |
| Uncomfortable with the degree of anonymity | ⁻ | ⁻ |
| Prefer face-to-face help | 2 | 8.3 |
|  |  |  |
| **Coach-related** |  |  |
| No support by coach | ⁻ | ⁻ |
| Different goals than coach | ⁻ | ⁻ |
| No connection with coach | ⁻ | ⁻ |
| Coaching is unnecessary | ⁻ | ⁻ |
| Did not like the form of coaching (text messages) | ⁻ | ⁻ |

**Table S4:** Module evaluation in detail

|  | | **Module 1** | **Module 2** | **Module 3** | **Module 4** | **Module 5** |
| --- | --- | --- | --- | --- | --- | --- |
| **Evaluation criteria** | | (n = 41) | (n = 27) | (n = 21) | (n = 12) | (n = 11) |
|  |  |  |  |  |  |  |
| General usefulness | | 7.51/10 | 7.48/10 | 6.86/10 | 7.46/10 | 6.64/10 |
| Goals of module clearly defined | |  | | | | |
|  | Yes, n (%) | 38 (92.7) | 25 (92.6) | 19 (90.5) | 10 (83.3) | 8 (72.7) |
|  | Partly, n (%) | 3 (7.3) | 1 (3.7) | 2 (9.5) | 2 (16.7) | 3 (27.3) |
|  | No, n (%) | 0 | 1 (3.7) | 0 | 0 | 0 |
| Clear and easy to understand content | |  | | | | |
|  | Yes, n (%) | 41 (100) | 26 (96.3) | 18 (85.7) | 9 (75.0) | 10 (90.9) |
|  | Partly, n (%) | 0 | 1 (3.7) | 3 (14.3) | 3 (25.0) | 1 (9.1) |
|  | No, n (%) | 0 | 0 | 0 | 0 | 0 |
| Easy to navigate | |  | | | | |
|  | Yes, n (%) | 40 (97.6) | 25 (92.6) | 18 (85.7) | 10 (83.3) | 9 (81.8) |
|  | Partly, n (%) | 1 (2.4) | 2 (7.4) | 3 (14.3) | 2 (16.7) | 2 (18.2) |
|  | No, n (%) | 0 | 0 | 0 | 0 | 0 |
| Appropriate length of the module | |  | | | | |
|  | Yes, n (%) | 34 (82.9) | 25 (92.6) | 17 (81.0) | 10 (83.3) | 8 (72.7) |
|  | Partly, n (%) | 7 (17.1) | 2 (7.4) | 3 (14.3) | 2 (16.7) | 3 (27.3) |
|  | No, n (%) | 0 | 0 | 1 (4.7) | 0 | 0 |
| Useful illustrations, pictures and figures | |  | | | | |
|  | Yes, n (%) | 28 (68.3) | 14 (51.9) | 11 (52.4) | 8 (66.7) | 3 (27.3) |
|  | Partly, n (%) | 11 (26.8) | 8 (29.6) | 8 (38.1) | 3 (25.0) | 7 (63.6) |
|  | No, n (%) | 2 (4.9) | 1 (3.7) | 2 (9.5) | 1 (8.3) | 1 (9.1) |
| Use of clear language, idiom and words | |  | | | | |
|  | Yes, n (%) | 40 (97.6) | 25 (92.6) | 19 (90.5) | 11 (91.7) | 10 (90.9) |
|  | Partly, n (%) | 1 (2.4) | 2 (7.4) | 2 (9.5) | 1 (8.3) | 1 (9.1) |
|  | No, n (%) | 0 | 0 | 0 | 0 | 0 |
| *Note.* General usefulness of the module was rated on a scale of 1 (not useful at all) to 10 (very much useful). | | | | | | |

| **Module 1** | **Module 2** | **Module 3** | **Module 4** | **Module 5** |
| --- | --- | --- | --- | --- |
| **What did you like about the module?**   - Scientific facts easily explained - Step by step - Basics - Clear - Informative - Goal-setting - Helpful | **What did you like about the module?**   - Informative - New exercise: Sleep restriction - Clear explanation - Specific instructions - Warning about task difficulty - Clip for extra explanation | **What did you like about the module?**   - Practical tools - Concrete exercises - Useful and relevant | **What did you like about the module?**   - Clear explanation - Practical aspect: Evaluating thinking errors myself - GGG schema | **What did you like about the module?**   - Good summary - Indicating that one can continue with the exercises |
| **What did you dislike about the module?**   - Much information - Very long - Difficult for students to separate the bedroom from the study - Not the right solution for everything - Obvious information | **What did you dislike about the module?**   - Daily sleep reporting - Not fitting my sleep problem - Bad timing - Not manageable due to exams - Some instructions are less clear | **What did you dislike about the module?**   - Lack of clarity on when to do relaxation exercises - Lack of explanation on how this will improve sleep quality | **What did you dislike about the module?**   - Focus on insomnia (oversleeping can also be addressed with these methods) | **What did you dislike about the module?** |
| **What would you like to see improved?**   - What tips to prioritize? - Improve language tone in lifestyle part - Split module in two - Reminder to finish module - Login where left off - Make more interactive - Include more practical tips & specific techniques | **What would you like to see improved?**   - Provide overview of the exercises before starting the program - Split the module for different types of sleep problems - Less frequent sleep reporting - Make more interactive | **What would you like to see improved?**   - Need for subtitles/transcript in exercises - Information on what to do if sleep worsened after sleep restrictions. | **What would you like to see improved?**   - Module 3 and 4 make sense before the sleep restriction - Use a general term like "sleep problem" instead of "insomnia" - More information on the benefits of the exercise | **What would you like to see improved?**   - Make a little more compact - Provide advice/tips to help you stick with doing the exercises - Summary of the previous modules could be a bit more concrete - Measure stressful life events |

**Table S5:** Module evaluations - Overview of textual feedback

**Table S6:** Items of the sleep and light exposure diary

| **#** | **Item** | **Question and explanation** |
| --- | --- | --- |
| 1 | Time of lights out | What time did you go to sleep?  This concerns the night of  Sunday (dd-mm-yyyy) to Monday (dd-mm-yyyy)  Explanation: Please indicate what time you went to sleep. This is the time when you turned off the light to go to sleep.  Example 1: you went to bed at 11:00 PM and turned the light off immediately. In this case you write down 11:00 PM.  Example 2: you went to bed but read for half an hour and turned off the lights at 11:30 PM. In this case you write down 11:30 PM.  Example 3: you went to bed and turned off the light at 23:00 PM but ended up falling asleep only at 01:00 AM. In this case you write 23:00. You can indicate the 2 hours of lying awake on the next page. |
| 2 | Time of getting up | What time did you get up?  This concerns the night of  Sunday (dd-mm-yyyy) to Monday (dd-mm-yyyy)  Explanation: Indicate here what time you got out of bed. This may be a different time than the time you woke up.  Maybe you woke up at 7:30 AM but got up at 8:00 AM. In this case, choose 8:00 AM here and continue on the next page. |
| 3 | Periods of being awake between time of lights out and time of getting up | Have you been lying awake?  Indicate when you were awake on the night of  Sunday (dd-mm-yyyy) to Monday (dd-mm-yyyy)  Explanation: Indicate here when you were awake during the night. You can also choose whether you went out of bed during the time you were awake at night or whether you stayed in bed. Select the boxes from the timetable by clicking. One box equals 15 minutes. |
| 4 | Times of napping | Did you take a nap?  Indicate when you took a nap on  Sunday (dd-mm-yyyy)  Explanation: A nap is the amount of time you slept during the day, whether you were in bed or not. Select the boxes from the timetable by clicking. One box equals 15 minutes.  You did not take a nap? Then skip this page and click on 'Next'. |
| 5 | Times of being outside | Have you been outside?  Indicate when you went outside on  Sunday (dd-mm-yyyy)  Explanation: Indicate when you have been outside the house. These are all the moments when you have not had a roof over your head.  You have not been outside? Then skip this page and click on 'Next'. |
| 6 | Screen use before sleeping | Screen use before sleeping?  This concerns the evening of  Sunday (dd-mm-yyyy)  Yes/No  Explanation: Did you use a screen an hour before going to sleep (such as a mobile phone, tablet, e-reader, laptop, or TV)? Only indicate "Yes" if you have looked at the screen for at least 5 minutes. Indicate "No" if you have only set an alarm clock. |
| 7 | Feeling of being rested | How rested do you feel this morning?  This concerns the night of  Sunday (dd-mm-yyyy) to Monday (dd-mm-yyyy)  Scale of 0 (not at all rested) to 10 (very much rested) |
| 8 | Sleep quality | How would you rate the quality of your sleep?  This concerns the night of  Sunday (dd-mm-yyyy) to Monday (dd-mm-yyyy)  Scale of 0 (very badly) to 10 (very well)  Explanation: Quality of sleep is your own feeling of whether you have slept well or badly. |

**Table S7:** Evaluation Sleep and Light Exposure Diary in detail

|  | **Strongly disagree (n, %)** | **Disagree (n, %)** | **Neutral (n, %)** | **Agree  (n, %)** | **Strongly agree  (n, %)** |
| --- | --- | --- | --- | --- | --- |
| 1. I think I would like to use the sleep diary more often | 2 (7%) | 3 (11%) | 9 (32%) | 10 (36%) | 4 (14%) |
| 2. I found the sleep diary unnecessarily complex | 20 (71%) | 2 (7%) | 5 (18%) | 1 (4%) | 0 |
| 3. I thought the sleep diary was easy to navigate | 0 | 3 (11%) | 4 (14%) | 6 (21%) | 15 (54%) |
| 4. I can imagine that most people would learn to use the sleep diary very quickly | 1 (4%) | 2 (7%) | 4 (14%) | 7 (25%) | 14 (50%) |
| 5. I found the explanation of the sleep diary clear | 0 | 0 | 3 (11%) | 11 (39%) | 14 (50%) |
| 6. I found the results of the sleep diary (the graphs) easy to understand | 0 | 0 | 5 (18%) | 10 (36%) | 13 (46%) |
| 6a. The graph about the average hours of sleep was clear | 0 | 0 | 3 (11%) | 10 (36%) | 15 (54%) |
| 6b. The graph about the average minutes awake was clear | 0 | 0 | 5 (18%) | 9 (32%) | 14 (50%) |
| 6c. The graph about the sleep efficiency was clear | 0 | 0 | 7 (25%) | 8 (29%) | 13 (46%) |
| 7. I understand what is meant by 'sleep efficiency' | 0 | 3 (11%) | 4 (14%) | 6 (21%) | 15 (54%) |
| 8. It takes too long to fill out the sleep diary | 11 (39%) | 0 | 7 (25%) | 9 (32%) | 1 (4%) |

**Table S8**: Questionnaire outcomes in intervention initiators

| **Measure** | **N** | **Pre-test, mean (SD)** | **Post-test, mean (SD)** | ***p* value** | **Cohen's *d* (95% CI)** |
| --- | --- | --- | --- | --- | --- |
|  |  |  |  |  |  |
| **ISI**  Insomnia Severity | 27 | 15.15 (4.08) | 9.15 (4.83)* | **<0.001** | 1.34 (0.74, 1.93) |
| **PHQ-9** Depression | 24 | 10.38 (5.13) | 7.08 (4.95)* | **<0.001** | 0.65 (0.07, 1.23) |
| **GAD-7** Anxiety | 24 | 7.63 (6.12) | 5.50 (4.94)* | **0.006** | 0.38 (-0.19, 0.95) |
| **WSAS** Functioning | 24 | 16.96 (7.68) | 12.50 (9.10)* | **0.038** | 0.53 (-0.05, 1.10) |
| **MHQoL** Quality of life | 24 | 13.21 (3.75) | 13.84 (3.46) | 0.139 | -0.17 (-0.74, 0.39) |
| **MHQoL** Overall mental wellbeing | 24 | 5.75 (2.09) | 6.29 (1.65) | 0.102 | -0.29 (-0.85, 0.28) |
| **MCTQ** Chronotype (mid sleep, local time) | 10 | 04:16 (02:24) | 04:41 (00:40) | 0.579 | -0.23 (-1.11, 0.65) |
| **MCTQ**  Average Sleep Duration | 24 | 6 h 54 min (1h 50 min) | 8 h 11 min (56 min)* | **0.004** | -0.48 (-1.01, 0.04) |
| **MCTQ** Absolute Social Jetlag | 24 | 48 min (35 min) | 56 min (44 min) | 0.494 | -0.04 (-0.48, 0.55) |
|  |  |  |  |  |  |
| *Note.* ISI: Insomnia Severity Index: 7-item insomnia scale; scores ranging from 0 - 28; higher scores indicate higher insomnia severity. PHQ-9: 9-item Patient Health Questionnaire; scores ranging from 0-27; higher scores indicate higher depression severity. GAD-7: 7-item Generalized Anxiety Disorder scale; scores ranging from 0-21; higher scores indicate higher anxiety severity. WSAS: 5-item Work and Social Adjustment scale; scores ranging from 0-40; higher scores indicate higher impairment in functioning. MHQoL: 8-item Mental Health Quality of Life Questionnaire; scores ranging from 0-21; higher scores indicate better quality of life. MHQoL Overall mental wellbeing rated on a scale of 1 (worst possible wellbeing) to 10 (best possible wellbeing). MCTQ: 17-item Munich Chronotype Questionnaire; self-report sleep measure comparing sleep outcomes on work/study days versus free days. h: hours; min: minutes; MCTQ chronotype in local time. | | | | | |
| **p* < 0.05, statistically significant difference to pre-test | | |  |  |  |

**Figure 3:** Distribution of responses on the separate ISI items in all included participants (n= 101)


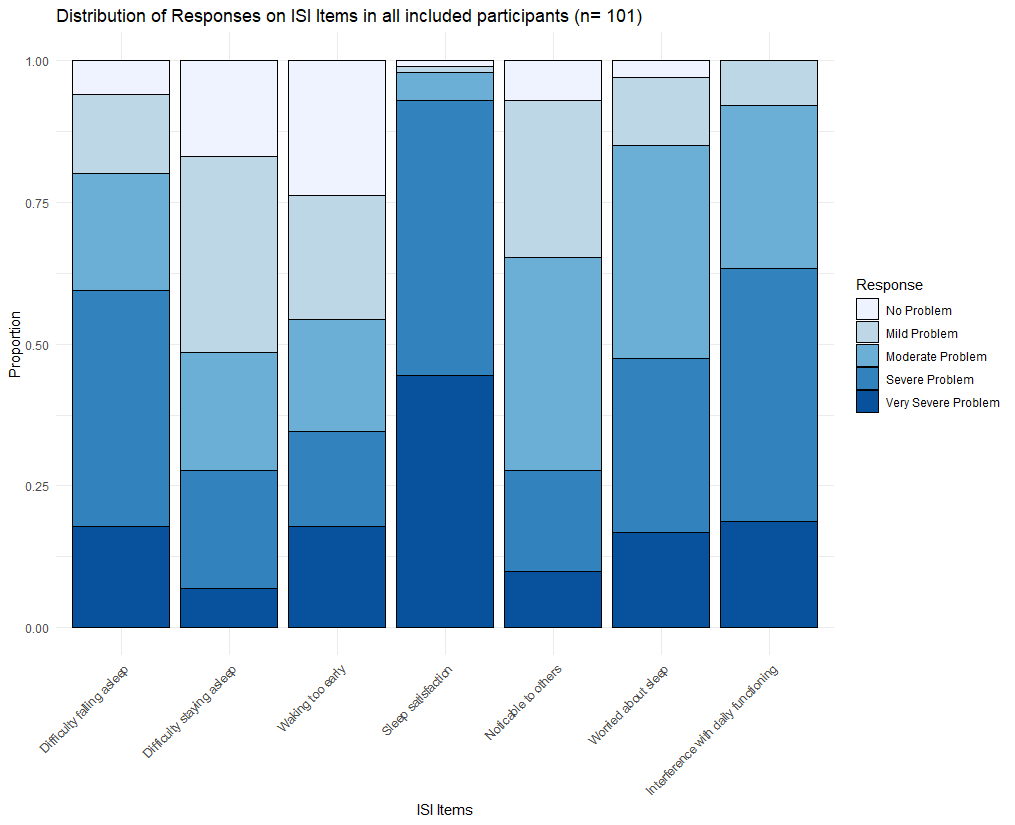


**Table 9:** Sleep and light exposure diary outcomes in intervention initiators (n= 41)

| **Outcome** | | **B** | **SE** | **t-value** | **p-value** | **B** | **95%CI** | **z-value** | **p-value** |
| --- | --- | --- | --- | --- | --- | --- | --- | --- | --- |
| **TST** | |  |  |  |  |  |  |  |  |
|  | Intercept | 436.97 | 10.27 | 42.54 | <.001* |  |  |  |  |
|  | Time | 0.30 | 0.12 | 2.50 | .012* |  |  |  |  |
| **Sleep quality** | |  |  |  |  |  |  |  |  |
|  | Intercept | 5.64 | 0.19 | 30.49 | <.001* |  |  |  |  |
|  | Time | 0.01 | 0.003 | 2.42 | .02* |  |  |  |  |
| **Refreshed** | |  |  |  |  |  |  |  |  |
|  | Intercept | 5.05 | 0.20 | 25.04 | <.001* |  |  |  |  |
|  | Time | 0.01 | 0.003 | 3.13 | .002 |  |  |  |  |
| **SE** | |  |  |  |  |  |  |  |  |
|  | Intercept | 0.62 | 0.26 | 29.54 | <.001* |  |  |  |  |
|  | Time | 0.001 | 0.00 | 4.37 | <.001* |  |  |  |  |
| **Total LE** | |  |  |  |  |  |  |  |  |
|  | Intercept | 43.30 | 0.26 | 12.88 | <.001* |  |  |  |  |
|  | Time | 0.0001 | 0.00 | -2.17 | .03* |  |  |  |  |
| **SOL** | |  |  |  |  |  |  |  |  |
|  | Intercept |  |  |  |  | 1.53 | (1.08 - 2.18) | 2.38 | .017* |
|  | Time |  |  |  |  | 0.99 | (0.99 - 1.00) | -2.47 | .013* |
| **WASO** | |  |  |  |  |  |  |  |  |
|  | Intercept |  |  |  |  | 0.97 | (0.66– 1.42) | -0.16 | 0.87 |
|  | Time |  |  |  |  | 0.99 | (0.99 – 1.00) | -2.53 | .012* |
| **EMA** | |  |  |  |  |  |  |  |  |
|  | Intercept |  |  |  |  | 1.04 | (0.71 – 1.52) | 0.21 | 0.83 |
|  | Time |  |  |  |  | 0.99 | (0.98 – 0.99) | -5.33 | <.001* |

*Note*. Analysis of intervention initiators (who initiated the main modules and provided sleep diary data). Results of linear mixed models depicted in left column. Results of negative binominal mixed models depicted in right column. TST = Total sleep time in minutes; Sleep quality = ‘*How did you sleep last night?*’ Scores ranging from 0 = *not good at all* to 10 *very good*); Refreshed = ‘*How refreshed are you feeling this morning?*’ Scores ranging from 0 = *not at all* to 10 *very much*); SE = Sleep Efficiency in %; Total LE = Total daylight exposure in minutes; SOL = Sleep Onset Latency in minutes; WASO = Wake After Sleep Onset in minutes. EMA = Early morning awakening in minutes. Tukey transformed estimates are shown for sleep efficiency. The unit for the outcome variable SOL and WASO were 15 minutes intervals recoded into levels. The Estimate and the 95% Confidence Intervals are calculated for the exponentiated results.

**Appendix: Caring Universities Consortium Scientific Committee Members**

The following are the members of the scientific committee of the Caring Universities Consortium, listed by their affiliated institution. This committee consists of one or more senior researchers from each academic consortium partner:

Vrije Universiteit Amsterdam:
Sascha Struijs

Leiden University:
Nadia Garnefski, Vivian Kraaij

Utrecht University:
Elske Salemink

Maastricht University:
Petra Hurks

Erasmus University Rotterdam:
Marilisa Boffo, Danielle Remmerswaal

University of Amsterdam:
Reinout Wiers, Claudia van der Heijde

InHolland University of Applied Sciences:
Lisa Klinkenberg

Rotterdam University of Applied Sciences:
Monique de Bruijn-Smolders

Avans University of Applied Sciences:
Jessica Nooij
